# Ultra-Processed Food vs. Fruit and Vegetable Consumption before and during the COVID-19 Pandemic among Greek and Swedish Students

**DOI:** 10.3390/nu15102321

**Published:** 2023-05-16

**Authors:** Friska Dhammawati, Petter Fagerberg, Christos Diou, Ioanna Mavrouli, Evangelia Koukoula, Eirini Lekka, Leandros Stefanopoulos, Nicos Maglaveras, Rachel Heimeier, Youla Karavidopoulou, Ioannis Ioakimidis

**Affiliations:** 1 Innovative Use of Mobile Phones to Promote Physical Activity and Nutrition Across the Lifespan (the IMPACT) Research Group, Department of Biosciences and Nutrition, Karolinska Institutet, 14152 Stockholm, Sweden; 2 Department of Informatics and Telematics, Harokopio University of Athens, 177 78 Athens, Greece; 3 Ekpaideftiria N. Mpakogianni, 415 00 Larissa, Greece; 4 2nd Obstetrics and Gynaecology Dept, Center of Woman Digital Health, Medical School, Aristotle University of Thessaloniki, 541 24 Thessaloniki, Greece; 5 Image and Video Processing Laboratory, Department of Electrical and Computer Engineering, Northwestern University, Evanston, IL 60208, USA; 6 Internationella Engelska Gymnasiet, 17164 Stockholm, Sweden; 7 Laboratory of Medical Informatics, Aristotle University of Thessaloniki, 541 24 Thessaloniki, Greece

**Keywords:** COVID-19 pandemic, children, dietary behaviors, ultra-processed food, main meals, lunch, dinner

## Abstract

Background: The COVID-19 pandemic has impacted children’s lifestyles, including dietary behaviors. Of particular concern among these behaviors is the heightened prevalence of ultra-processed food (UPF) consumption, which has been linked to the development of obesity and related non-communicable diseases. The present study examines the changes in (1) UPF and (2) vegetable and/or fruit consumption among school-aged children in Greece and Sweden before and during the COVID-19 pandemic. Methods: The analyzed dataset consisted of main meal pictures (breakfast, lunch, and dinner) captured by 226 Greek students (94 before the pandemic and 132 during the pandemic) and 421 Swedish students (293 before and 128 during the pandemic), aged 9–18, who voluntarily reported their meals using a mobile application. The meal pictures were collected over four-month periods over two consecutive years; namely, between the 20th of August and the 20th of December in 2019 (before the COVID-19 outbreak) and the same period in 2020 (during the COVID-19 outbreak). The collected pictures were annotated manually by a trained nutritionist. A chi-square test was performed to evaluate the differences in proportions before versus during the pandemic. Results: In total, 10,770 pictures were collected, including 6474 pictures from before the pandemic and 4296 pictures collected during the pandemic. Out of those, 86 pictures were excluded due to poor image quality, and 10,684 pictures were included in the final analyses (4267 pictures from Greece and 6417 pictures from Sweden). The proportion of UPF significantly decreased during vs. before the pandemic in both populations (50% vs. 46%, *p* = 0.010 in Greece, and 71% vs. 66%, *p* < 0.001 in Sweden), while the proportion of vegetables and/or fruits significantly increased in both cases (28% vs. 35%, *p* < 0.001 in Greece, and 38% vs. 42%, *p* = 0.019 in Sweden). There was a proportional increase in meal pictures containing UPF among boys in both countries. In Greece, both genders showed an increase in vegetables and/or fruits, whereas, in Sweden, the increase in fruit and/or vegetable consumption was solely observed among boys. Conclusions: The proportion of UPF in the Greek and Swedish students’ main meals decreased during the COVID-19 pandemic vs. before the pandemic, while the proportion of main meals with vegetables and/or fruits increased.

## 1. Introduction

Childhood overweight and obesity are serious global public health concerns [1]. In childhood, excess body weight has been strongly linked with several non-communicable diseases and future morbidity, in addition to causing negative effects on psychological well-being and school performance [2,3]. The growing sales and consumption of ultra-processed food (UPF) appears to be a significant contributing factor to global obesity development [4]. UPFs are typically defined as industrially manufactured products made primarily from food substances with minimal whole foods. The NOVA classification system designates UPFs as Group 4, indicating that they have undergone substantial processing, which results in lower nutritional quality compared to minimally processed or whole foods [5]. Nutritional analysis has shown that UPFs tend to be high in energy density and contain high amounts of added sugar, salt, and/or saturated fatty acids, but they are low in dietary fiber, micronutrients, and phytochemicals, making them less healthy than minimally processed or whole foods [6]. UPF manufacturing aims to create highly palatable products that are convenient to consume, have long shelf lives, are inexpensive, and easily accessible [7]. The shift in dietary patterns towards increased UPF consumption has been associated with numerous health problems, including obesity, type 2 diabetes, cardiovascular diseases, and some types of cancer [8]. Numerous studies have shown that UPFs are associated with a higher risk of overweight and obesity [9]. Moreover, a well-designed randomized controlled trial demonstrated that individuals who consumed a UPF diet experienced a significant increase in energy intake and body weight gain compared to those who followed an unprocessed diet [10]. 

More recently, the occurrence of COVID-19, which was officially declared a pandemic by the World Health Organization (WHO) on 11 March 2020, has affected and altered the lives of people around the globe [11]. The closure of educational institutions in most countries, as one of the strategies to reduce the risk of viral spreading, has subsequently affected children’s lifestyles [12,13]. Some studies have reported changes in children’s dietary behaviors, including a decrease in fast-food consumption and an increase in self-reported consumption of legumes, fruits, and vegetables by children and adolescents [14,15]. However, other studies have suggested an increase in the intake of non-nutritive foods, such as sweet and high-calorie snack foods, as reported by parents [16]. However, the knowledge of the impact of the COVID-19 pandemic on children’s dietary behaviors is still limited [17].

The present study aims to investigate changes in dietary habits among school-aged children in Greece and Sweden, independently, within each of the countries, before and during the COVID-19 pandemic. The analysis is based on food content annotated in main meal pictures (i.e., breakfasts, lunches, and dinners) submitted by the children themselves. The presented data were collected over four-month periods over two consecutive years; namely, between the 20th of August and the 20th of December in 2019 (before the COVID-19 outbreak) and the same period in 2020 (during the COVID-19 outbreak). The analysis focused on examining the proportion of meals containing: (i) UPF and (ii) fruits and/or vegetables in each period within each country. It is important to note that the students were not required to modify their dietary habits or eating behaviors in any way during the study. The collection of meal pictures using a mobile application allowed for the accurate and objective assessment of the students’ dietary behaviors regarding their main meals before and during the pandemic.

## 2. Materials and Methods

### 2.1. Study Design 

**Figure 1:**
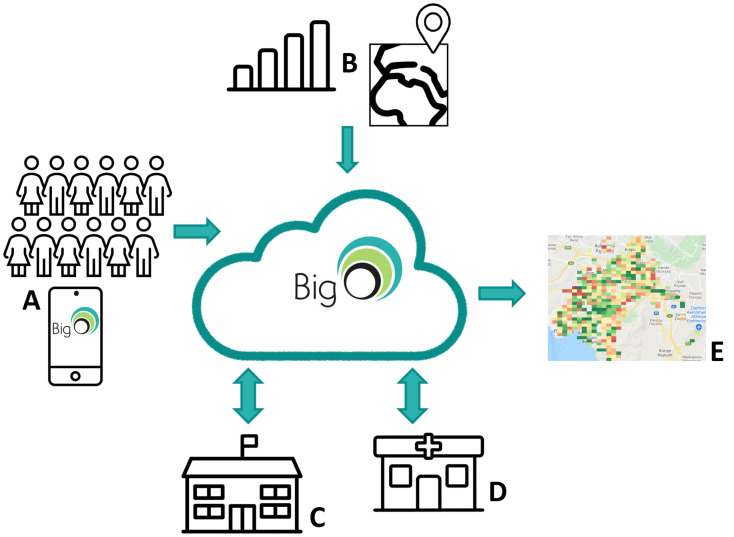
Big Data Against Childhood Obesity (BigO) project overview. (**A**) A cohort of children was recruited through Schools (**C**) and Clinics (**D**), using the myBigO app (**A**), contributed lifestyle and living environment data. Using parallel input from open-access national statistics and maps (**B**), the provided datasets were analyzed in the BigO Cloud and were used to support education in schools (**C**) and patient monitoring in clinics (**D**), while they were also aggregated and were made available to local Public Health Authorities through a dedicated visualization portal (**E**) [20].

This study used data from the European project Big Data against Childhood Obesity (BigO; Figure 1). Briefly, the BigO project focused on creating technological/scientific tools to collect and analyze data on children’s behavioral patterns and living environments [18]. Details about the project are published elsewhere [19]. A periodic, cross-sectional, multicentric study design was applied to investigate the difference in the proportion of UPF and fruit and/or vegetable consumption for the main meals (breakfast, lunch, and dinner) before and during the COVID-19 pandemic among the Swedish and Greek school-aged children.

### 2.2. Setting

The meal pictures were collected by students in Ekpaidefitria N. Bakogiannis S.A. school in Larissa, Greece, and in Internationella Engelska Gymnasiet in Stockholm, Sweden for two consecutive years (2019 and 2020) using the myBigO app [20]. The recruitment was not a random selection of schools in Sweden and Greece; the participating schools were BigO project partners, having active data-collection actions before and during the pandemic outbreak. The research site in Sweden is a dual-curriculum high school in the Stockholm metropolitan area with about 700 students of mixed backgrounds. The research site in Greece is a privately run school in the Larissa city area in central Greece. The meal pictures were collected between 20 August and 20 December 2019 and represented the children’s dietary behaviors before the COVID-19 pandemic, while pictures collected between the same dates in 2020 represented the children’s dietary behaviors during the COVID-19 pandemic.

### 2.3. Participants

The participants in this study were Swedish and Greek students with ages ranging from 9 to 18 years. The recruitment of participants was conducted in a spontaneous, non-discriminative, and all-inclusive fashion. All students in Ekpaidefitria N. Bakogiannis S.A., Larissa, Greece, and Internationella Engelska Gymnasiet, Stockholm, Sweden, were invited to participate. No inclusion nor exclusion criteria were applied other than being willing to take part in the study procedures and providing informed consent, as well as being part of the included schools. Due to the cross-sectional nature of the data collection, the students participating before and during the COVID-19 pandemic were not the same individuals.

### 2.4. Data Collection and Procedure

The students who were participating in the BigO project took pictures of their main meals with the myBigO app on their personal smartphones [20]. The myBigO app was developed by the BigO project team specifically for students to collect information about their lifestyle, health behaviors, and the environment they live in. The app collects data passively, via the use of accelerometer and GPS sensors of the mobile phones, as well as actively, by asking students to answer questions and take pictures [18]. The passively collected data were not used in this study. To anonymize the data, students first had to choose a nickname in the app; afterward, they were asked to input their gender, height, weight, age, and city of residence. To upload a picture, they had to select a simple classification for the recorded meal (breakfast, lunch, dinner, snack, or drink). After selecting one of those options, the app opened the camera to allow the user to take the picture and then submit it to the BigO server.

All the pictures submitted by the students were manually reviewed by BigO personnel to ensure that the pictures did not contain anything that could reveal the identity or personal information of the children [17]. Upon voluntary enrolment in the BigO data collection, the students were uniformly given instructions to submit as many meal/drink pictures as they could, including at least 2 main meals and 3 snacks per day for a period of at least two weeks. However, since the data collection was uncontrolled, the participating students were eventually free to upload fewer or more pictures.

### 2.5. Ultra-Processed Food

Foods categorized as UPFs, as defined in the NOVA categorization system, are industrially manufactured food products consisting mostly or entirely of substances that are not normally used in culinary preparations [6]. Some examples of UPFs are fries, reconstituted meat products, commercial sauces, ready-to-heat meals, dehydrated soups and noodles, sausages, burgers, industrialized bread, breakfast cereals, etc. In the present study, the meal was annotated as UPF when at least 1/4 cup of UPF in solid form or 2 tablespoons in liquid form (such as sauces and condiments) were observed in the picture. 

### 2.6. Vegetables and Fruits

The list of fruits and vegetables on the Swedish National Food Agency website was used as a reference in annotating the fruits and vegetables [21] when at least 1/3 cup of fruits/berries or vegetables were visible in the picture. For analysis homogeneity purposes, this was the case for both countries’ datasets.

### 2.7. Picture Analysis Process

Before annotating the meal pictures, the nutritionist (F. D.) was trained by a senior researcher, firstly by studying the meaning and the general characteristics of UPF and recognizing some examples of UPFs referring to some published articles [6,10,22,23,24]. Afterward, one hundred meal pictures from the dataset were randomly drawn and annotated independently by the rater and the nutritionist. The annotations were compared, and the results were used to identify major disagreements related to the UPF and fruit/vegetable categories, using those as examples in the training of the rater. Note that this was a training procedure, not connected with the reliability analysis that is presented below.

**Figure 2:**
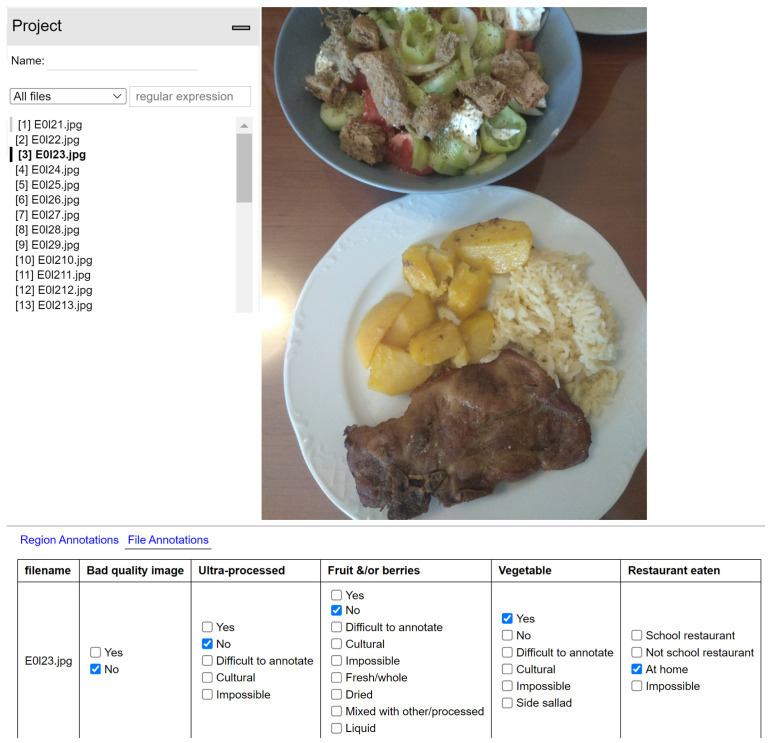
Screenshot of the meal picture annotating procedure using VIA.

To construct the research dataset, all the submitted pictures from the selected schools for the specific time periods, categorized by the students themselves as “main meals” (breakfast, lunch, and dinner, categorized in the BigO app), were downloaded from the BigO server (10,770 pictures in total out of more than 120,000 pictures that had been collected by the BigO project). All the pictures (originating from Greece or Sweden, before or during the Pandemic) were placed in a common repository. The filename, the nickname of the contributing student, and the country, school, and date information of all the pictures were removed, and the order of the pictures was randomized by an independent researcher using a custom Excel script to blind the rater. The final picture analysis and categorization were performed using the picture annotation program VGG Image Annotator (VIA) [25]. The presence of UPF, fruits, and vegetables in the meal pictures was annotated accordingly (Figure 2). 

**Figure 3:**
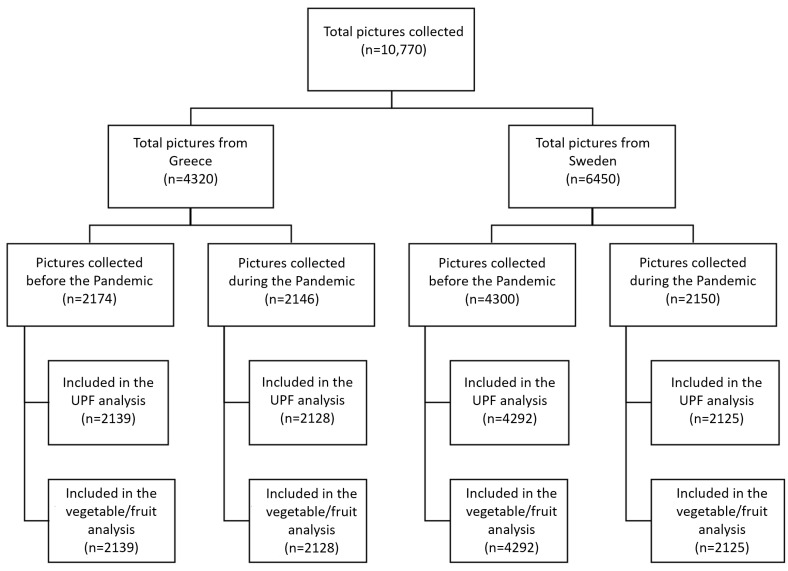
Number of pictures analyzed in the study.

Some pictures were excluded from the analysis if they were too blurry or too dark, making it impossible to interpret the food content. Other pictures were excluded if the rater could not identify food content relevant to a main meal (e.g., chewing gum was photographed). The final number of pictures included in the analysis can be seen in Figure 3.

### 2.8. Inter-Rater Agreement

To test the inter-rater agreement for the annotations, 1000 pictures from the final dataset were randomly selected and annotated independently by the rater and a researcher. Cohen’s Kappa test was conducted to measure the inter-rater agreement of the meal picture annotations. Kappa value 0–0.20 was interpreted as “No agreement”; 0.21–0.39 “Minimal agreement”; 0.40–0.59 “Weak agreement”; 0.60–0.79 “Moderate agreement” 0.80–0.90 “Strong agreement”; and above 0.90 as “Almost perfect agreement” [26].

### 2.9. Data Analysis

Meal pictures containing fruits were merged and analyzed as one category with the meal pictures containing vegetables. Meanwhile, the annotation analysis of the UPF category was performed independently. A chi-square test was performed to evaluate the proportion difference between the UPF and fruit and vegetable categories among the Greek students, as well as among the Swedish students before the pandemic and during the pandemic. The differences in mean age, BMI, and BMIz before and during the COVID-19 pandemic were analyzed using an independent *t*-test. SPSS version 27 (IBM, Armonk, NY, USA) software [27] was used to perform all statistical analyses, and a significance level of *p* < 0.05 was applied to all statistical tests.

### 2.10. Ethics and Approval Consent to Participate

The collection of meal pictures among the Swedish students was approved by the Swedish Ethical Review Board (Etikprövningsnämnden) in Stockholm, DNR: 2018/1921-31/5, while the Committee for proper practice in Research (Επιτροπή Δεοντολογίας στην Έρευνα), Aristotle University of Thessaloniki, DNR: 132649/2017, and Greek Republic Ministry of Education DNR: 1484/N1, approved the collection of meal pictures among the Greek students. Written informed consent was obtained from all the students that were participating in this study, as well as from their parents or legal guardians. All procedures were in accordance with the Helsinki Declaration of 2013.

## 3. Results

### 3.1. Data Overview and Population Characteristics

In total, 4320 pictures from Greece and 6450 pictures from Sweden were annotated in this study. Out of those, 86 pictures were excluded from the analyses due to poor image quality (see Figure 3). A total of 647 students contributed pictures for this dataset, including 226 and 421 from Greece and Sweden, respectively (Table 1). Additional information about the data-contribution distributions for each user, per age group and per BMIz category can be found in Supplementary Materials Figures S1 and S2, respectively.

In Greece, 2174 pictures were contributed before and 2146 during the pandemic. Similarly, in Sweden, 4300 and 2150 pictures were contributed, respectively.

Regarding the anthropometric characteristics of the contributing student populations (Table 1), there was no significant difference in the mean BMIz or the mean BMI of the participants before and during the pandemic in both countries. In Sweden, there was no significant mean age difference before vs. during the pandemic, but in Greece, the mean age of the participants was higher during the pandemic (12.15 vs. 12.55 years; *p* = 0.006).

### 3.2. Proportion of Ultra-Processed Food in Main Meals

**Figure 4:**
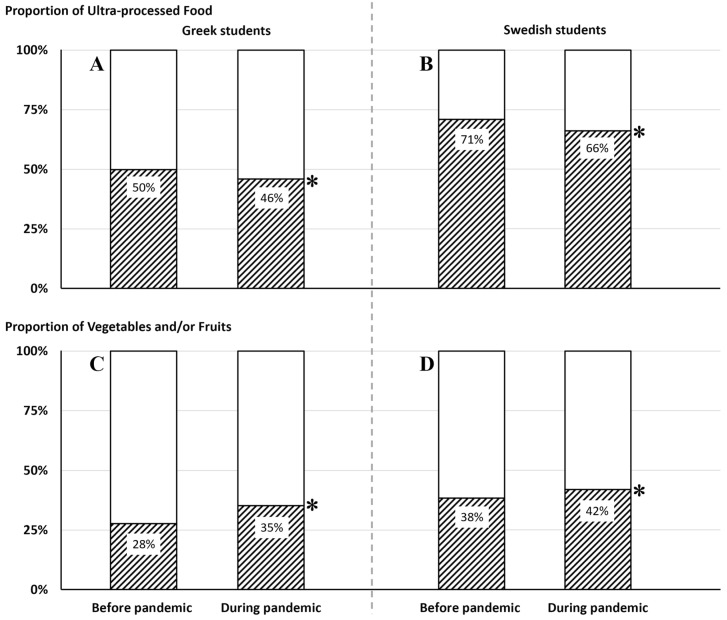
(**A**–**D**). Proportion of meal pictures containing UPF or Vegetables and/or Fruits before and during COVID-19 pandemic among Greek students (**A**,**C**) and Swedish students (**B**,**D**). * *p* < 0.05; χ^2^ comparison within country for respective food types between two consecutive years.

The proportion of meal pictures containing UPF was ~4% lower among the Greek students (50% before the pandemic vs. 46% during the pandemic, χ^2^ = 6.58, *p* = 0.010) and ~5% lower among the Swedish students (71% before the pandemic vs. 66% during the pandemic, χ^2^ = 15.44, *p* < 0.001) during the pandemic compared to before the pandemic. (Figure 4A,B and Table 2). The lower proportion of meal pictures containing UPF during the pandemic vs. before the pandemic was observed among boys in both Greece and Sweden, while among girls, there were no significant differences (Table 2).

### 3.3. Proportion of Vegetables and/or Fruits in Main Meals

The proportion of meal pictures containing vegetables and/or fruits during the pandemic increased ~7% among the Greek students (28% before the pandemic vs. 35% during the pandemic, χ^2^ = 28.25, *p* < 0.001) and ~4% among the Swedish students (38% before the pandemic vs. 42% during the pandemic, χ^2^ = 5.55, *p* = 0.019) compared to before the pandemic (Table 2). The increased proportion of meal pictures containing vegetables and/or fruits during the pandemic was observed among both boys and girls in the Greek sample, while in the Swedish sample, a significant increase was only observed among boys (Table 2).

### 3.4. Inter-Rater Agreement

The inter-rater agreement test for the annotation of the UPF category indicated a “weak-level agreement” between the annotator and the researcher (κ = 0.596). “Moderate-level agreement” was observed for the annotations of vegetables and/or fruit category (κ = 0.798).

## 4. Discussion

### 4.1. Summary of the Results

The dataset for the present study contained meal pictures collected by Greek and Swedish school-aged children for two consecutive years. The analysis of the children’s dietary behaviors initially showed a decrease in the proportion of meals containing UPF during the COVID-19 pandemic compared to before. Similarly, there was an increase in the proportion of vegetables and/or fruits contained in the students’ main meals during the pandemic. The proportional change in meal pictures containing UPF was observed among boys in both countries, while the increase in vegetables and/or fruits was observed for both genders in Greece and only among boys in Sweden. These results suggest that the UPF exposure was lessened, while the exposure to vegetables and/or fruits was increased during the COVID-19 pandemic.

### 4.2. Current Results in Relation to the Literature

Our results are consistent with the findings from prior studies that evaluated dietary changes among Greek children [28], Polish adolescents [15], and a multinational sample of adolescents [14], all showing favorable dietary changes during the COVID-19 pandemic. For example, a reduction in the consumption of fast foods and an increase in the intake of fruits and vegetables during the COVID-19 pandemic were self-reported by children and adolescents [14,28]. Similarly, there was an increase in the proportion of participants who declared that they had met the recommended intake of fruits (≥3 portions/day) and vegetables (≥4 portions/day) during the pandemic [15]. While there may be a disparity in the consumption of UPF and fruits/vegetables based on gender in both Greece and Sweden, this discrepancy is not considered statistically significant. However, both men and women in these countries have shown promising progress toward improving their diets during the pandemic, with a decrease in the consumption of UPF and an increase in the consumption of fruits and vegetables. Possible explanations for the shift in the children’s and adolescents’ dietary behaviors were the observed changes in health awareness, home food environments, exposure to fast-food restaurants, and child-feeding practices during the COVID-19 pandemic. Adolescents in Poland admitted that health motivation influenced their dietary choices to a higher degree during the pandemic compared to beforehand [29]. Additionally, another study reported concern among parents about their children’s overweight status and the subsequent use of restrictive feeding practices and diet monitoring during the COVID-19 pandemic [30]. 

However, it is important to note that the overall proportion of main meals containing UPFs in the current study was still high, despite the reductions observed during the pandemic. Results show that during the pandemic, 46% of the Greek children’s main meals still contained UPFs, while in the Swedish sample, the figure was as high as 66%. In fact, young populations have been reported as the leading consumers of UPF [31,32], presumably due to favorable taste and price, convenience, increased availability, and aggressive marketing [33]. Children themselves identify these factors as the main drivers of food choice [34,35]. Subsequently, early exposure to UPFs at a young age is potentially predisposing individuals to unhealthy dietary behaviors sustained throughout adulthood since taste expectations and preferences are shaped during childhood [36,37]. Ubiquitous UPF has been implicated in impairing individuals’ ability to regulate intake and weight, consequently resulting in overconsumption and weight gain [38]. Additionally, high consumption of UPF has been associated with lower consumption of fruits and vegetables [39]. Thus, an over-representation of UPFs in children’s meals is further contributing to children’s dietary challenges, given that children do not usually meet the recommendations for daily intake of fruits and vegetables [40,41]. 

Regarding the selection of the timepoint of this analysis, it should be noted that factors such as anxiety, fear, and increased insecurities caused by natural disasters and an unexpected situation, such as the COVID-19 pandemic, might result in acute dietary and everyday behavioral changes at the beginning of the pandemic [42]. However, the measurements during the pandemic were conducted about 5 months after the initiation of the pandemic (August–December 2020). Thus, the identified dietary effects are probably separate from the initial acute behavioral effects of the viral breakout and the sudden lifestyle changes. Instead, the study explored the children’s eating behavior that was established owing to the pandemic. Additionally, the same months and dates were chosen for both before and during the pandemic to control dietary variations due to the seasonal effects. Indeed, other studies have reported that variation in food items and nutrient intake is affected by seasonal changes [43], and this was especially noted for the intake of fat, groups of sugar, and fiber [44].

**Figure 5:**
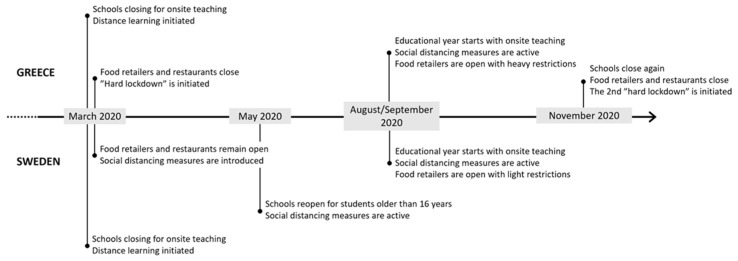
Timeline of COVID-related changes in schools’ and food retailers’ functions in Greece and Sweden during the first stage of the COVID-19 pandemic.

The time setting in this study further allowed an observation of the effect of temporary school closure during the pandemic on children’s eating behavior. During mid-November 2020 and at least until mid-January 2021, all primary schools, kindergartens, and daycares in Greece were closed [45,46], while schools in Sweden remained open [47]. Figure 5 summarizes the situation in both countries from the early stage of the outbreak to December 2020. 

Moreover, the two countries have different systems relating to the students’ school meals. Sweden is one of the few countries that provide cost-free and nutritious school meals for all students aged 7–16 [48], while Greek schools do not normally provide their students with school meals. Despite the discrepancy between the two systems, and the fact that the pandemic situation did not interrupt the Swedish students from having activities at school throughout the study period, similar trends of dietary changes related to UPF and vegetable and/or fruit consumption were found in both countries during this study.

### 4.3. Strengths and Limitations

The major strengths of the current study include the large sample size of meal pictures, real-time data collection, and observations from two consecutive years. The utilization of mHealth methodologies allowed for the real-time data collection and the serendipitous remote investigation of the children’s eating behaviors due to the COVID-19 pandemic situation despite the social mobility restrictions that were in effect. The data-collection method was also appropriate for the monitored population, as children reported their dietary intake using smartphone photos, which is suitable for the young population [49]. Such a method of population-level dietary assessment is more memory-independent vs. traditional questionnaire methods (i.e., food frequency questionnaires and 24-h recalls) and does not require participants to categorize the type of food that they are eating [49]. Accordingly, using personal smartphones for the photo recording of meals not only reduces the recall bias but also reduces the participant burden while, at the same time, it improves food identification, given that it is a trained observer who identifies and categorizes the food in each picture.

Several limitations of this study should be addressed. Correct meal content analysis, especially for the UPF category (i.e., by only looking at the meal pictures), is rather challenging due to the ambiguous nature of the photograph itself. The result of Cohen’s Kappa test on the UPF categorization fell within the weak-level agreement range (κ = 0.596). This was expected based on our previous agreement test (κ = 0.695) for this category in the context of food advertisements [33]. Subjective errors and biases when annotating the meal pictures, the somewhat ambiguous nature of the UPF category and the threshold amount for labeling the food as UPF, content misinterpretation, lack of knowledge due to the cultural gap (i.e., Swedish vs. Greek food culture in this study), and the obscurity of identifying whether the food was prepared from scratch or using industrially made products were the major challenges in this study. However, such errors were minimized by setting up standard operating procedures and extensive rate training before the analysis of the final dataset, allowing for better standardization of the annotations. On another note, the cross-sectional nature of the data collection dictated that the students participating before the pandemic and during the pandemic were not the same individuals, thus decreasing the power of the performed analyses. Another limitation was that only main meals were analyzed in the current study. Potential differences in snack food intake were, therefore, not investigated and could have affected the outcomes of the study if such meals had been included. Future studies should, therefore, include complete data on food intake rather than only reporting results for main meals. Additionally, since the recruitment was not a random selection of schools in Sweden and Greece (the participating schools were BigO project partners, having active data-collection actions before and during the pandemic outbreak), the analyzed sample cannot be regarded as fully representative of the Swedish or the Greek student populations. It should also be emphasized that the nature of data collection resulted in skewed datasets with regards to the “per participant” distribution (see Supplementary Material Figure S1), something that is also reflected in the skewed data-contribution distributions for “per age” and “per BMIz category” (see Supplementary Material Figure S2). This, in turn, limited our ability to perform sub-group comparative analyses within each country (e.g., “per age” or “per BMIz category”). Furthermore, the amount of food consumed by the subjects was not recorded in this study, making it impossible to evaluate any meal portion size effect of the pandemic occurrence. The nature of the performed data collection does not allow for the evaluation of meal occurrence frequency in any of the examined periods of time. Ideally, in future studies, additional information about portion size and meal frequency would be a good complement to similar datasets, as well as weight/BMI changes among the participating students.

## 5. Implications for Research and Practice

The current study demonstrated a lower proportion of UPF consumption during main meals and a higher proportion of meals containing vegetables and/or fruits during the COVID-19 pandemic compared to the year before the pandemic among school-aged children in Sweden and Greece. The findings presented in this study can be used as a stimulus for further research considering the role of the availability of UPF and/or fruits and vegetables on the food choices of adolescents at home (i.e., the main life-space setting during the COVID-19 outbreak). Using these outcomes, better interventions targeting the family space and home food availability can be tailored, promoting a healthier lifestyle.

## Figures and Tables

**Table 1:** Sample characteristics and data-contribution measures for the Greek and Swedish student population.

	Greece		*p* Value *	Sweden		*p* Value *
	Before Pandemic	During Pandemic		Before Pandemic	During Pandemic	
Participant, *n* (%)	94 (42%)	132 (58%)		293 (70%)	128 (30%)	
Female gender, *n* (%) *	39 (41%)	58 (44%)		144 (49%)	74 (58%)	
Mean number of pictures submitted	23	16		15	16	
Median number of pictures submitted	14	7		9	13	
Age, years (SD)	12.15 (±1.09)	12.55 (±1.05)	0.006	14.35 (±2.26)	14.39 (±2.39)	0.872
BMI, kg/m^2^ (SD)	18.95 (±2.93)	19.79 (±6.06)	0.165	20.18 (±3.76)	20.16 (±3.75)	0.949
BMIz, mean (SD)	0.12 (±1.26)	0.18 (±1.46)	0.748	−0.02 (±1.1)	−0.05 (±1.16)	0.790

* statistical significance (*p* < 0.05); *t*-test for independent samples.

**Table 2:** Proportion of UPF category and fruit and/or vegetable before vs. during pandemic among the Greek and Swedish male and female students, as well as for the total sample of both male and female students in each country.

	Greece		*p* Value	Sweden		*p* Value
	Before Pandemic	During Pandemic		Before Pandemic	During Pandemic	
Ultra-processed, *n* (%)						
Total sample ^†^	1067 (50%)	978 (46%)	0.010 *	3046 (71%)	1406 (66%)	<0.001 *
Female ^‡^	715 (49%)	483 (45%)	0.071	1779 (68%)	902 (66%)	0.176
Male ^‡^	352 (52%)	495 (47%)	0.036 *	1267 (76%)	504 (67%)	<0.001 *
Containing fruits, berries, and/or vegetables, *n* (%)						
Total sample ^†^	591 (28%)	749 (35%)	<0.001 *	1648 (38%)	881 (42%)	0.019 *
Female ^‡^	442 (30%)	399 (38%)	<0.001 *	1127 (43%)	561 (41%)	0.202
Male ^‡^	149 (22%)	350 (33%)	<0.001 *	521 (31%)	320 (42%)	<0.001 *

n = number; * statistical significance in bold (*p* < 0.05); *t*-test for independent samples; ^†^ “Total sample” percentages refer to the proportion of total pictures containing the relevant food category within the respective analyzed period; ^‡^ “Gender-specific percentages” refer to the proportion of gen-der-specific pictures containing the relevant food category within the total gender-specific pictures within the respective analyzed period.

## Data Availability

The datasets used and/or analyzed during the current study are available from the corresponding author on reasonable request.

## Supplementary Materials

The following supporting information can be downloaded at: https://www.mdpi.com/article/10.3390/nu15102321/s1, Figure S1: Contribution of data per age of the participant across the whole dataset (GR and SWE); Figure S2: Contribution of data per age of the participant across the whole dataset (GR and SWE); Interpretation of cut-offs based on WHO ^1^: Severe thinness: below −2, Thinness: from −2 to −1, Normal weight: −1 to 1, Overweight: 1 to 2; Obesity: more than 2 [50].
