# Supplementary material for: Ultra-Processed Food vs. Fruit and Vegetable Consumption before and during the COVID-19 Pandemic among Greek and Swedish Students
Source: Nutrients. 2023 May 16;15(10):2321. doi: 10.3390/nu15102321 (PMC10222979; doi:10.3390/nu15102321)
Supplement: Supplementary file 1 [file nutrients-15-02321-s001.zip › nutrients-2319431-supplementary.pdf]

Supplementary material 2.

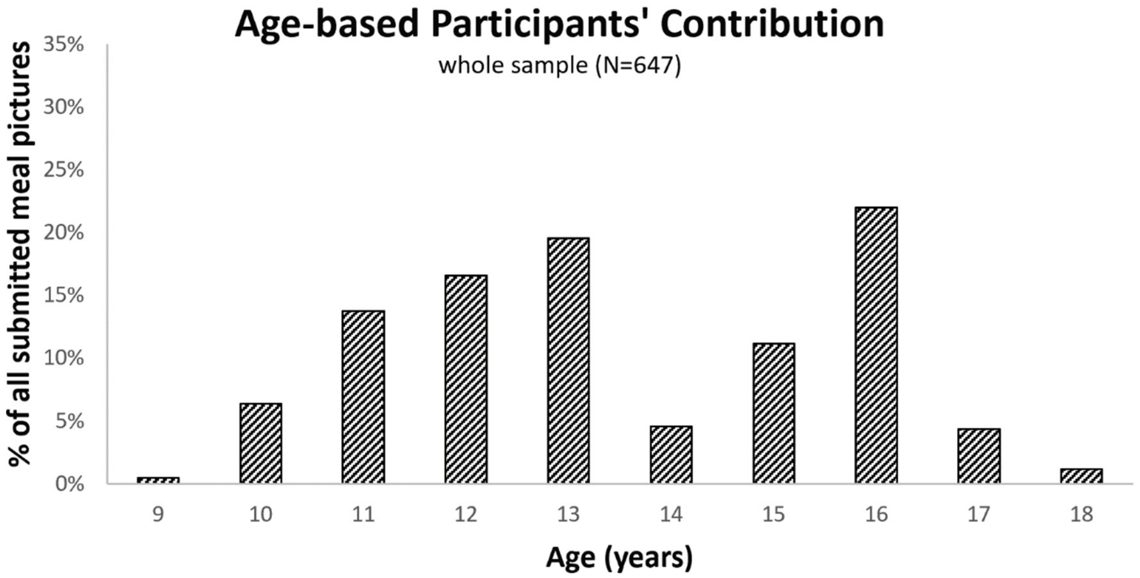

Figure S1: Contribution of data per age of the participant across the whole dataset (GR and SWE)

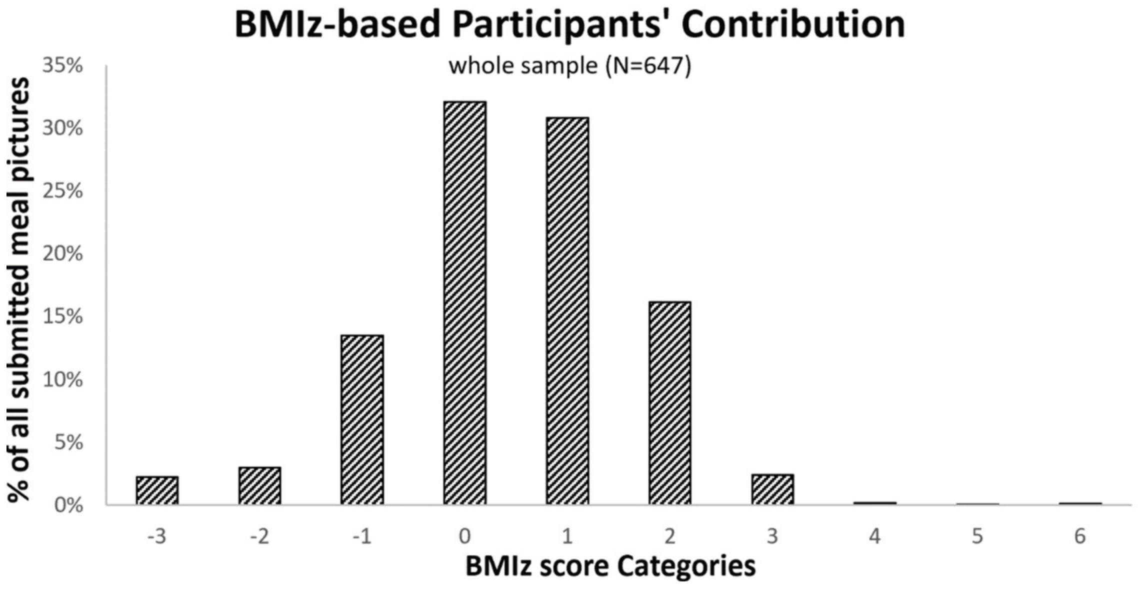

Figure S2: Contribution of data per age of the participant across the whole dataset (GR and SWE); Interpretation of cut-offs based on WHO[1]: Severe thinness: below -2, Thinness: from -2 to -1, Normal weight: -1 to 1, Overweight: 1 to 2; Obesity: more than 2.

References

1. “Growth Reference 5-19 Years - BMI-for-Age (5-19 Years).” *World Health Organization*, www.who.int/tools/growth-reference-data-for-5to19-years/indicators/bmi-for-age. Accessed 3 May 2023.
